# Supplementary material for: Improvement of Flowering Stage in Japonica Rice Variety Jiahe212 by Using CRISPR/Cas9 System
Source: Plants (Basel). 2024 Aug 5;13(15):2166. doi: 10.3390/plants13152166 (PMC11314265; doi:10.3390/plants13152166)
Supplement: Supplementary file 1 [file plants-13-02166-s001.zip › plants-3027683-supplementary.pdf]

Table S1. Primers used in this study.

Primer name	Primer sequence (5' —3')	Application
Hd2-g++	GGCAGCTCATCACAACCAAACCGC	Construction of knockout vectors
Hd2-g--	AAACGCGGTTTGGTTGTGATGAGC	
Ghd7-g++	GGCAATGTCGATGGGACCAGCAGC	
Ghd7-g--	AAACGCTGCTGGTCCCATCGACAT	
DTH8-g++	GGCACATCAGCTTCGTTACAGGCG	
DTH8-g--	AAACCGCCTGTAACGAAGCTGATG	
Pi21-g++	GGCATGCCAAGATCAGGAAGGTCC	
Pi21-g--	AAACGGACCTTCCTGATCTTGGCA	
Badh2-g++	GGCACAAGTACCTCCGCGCAATCG	
Badh2-g--	AAACCGATTGCGCGGAGGTACTTG	
Hd2-F	CTATTCTTTCTCATTTGTGAGATT	Sequencing identification of knockout targets
Hd2-R	AGAAGTGAGAAGGTAGAAGGAGGA	
Ghd7-F	GCCGAAGAACTGGAACTC	
Ghd7-R	GCCACTTCTAAGATCACACT	
DTH8-F	AAGGAGAGCATCACCACTCC	
DTH8-R	ACAGCATCAGCATCAACA	
Pi21-F	GAGCATGCCTGTTAATTGCAA	
Pi21-R	ATCTCCTTGATGATCTTGCCG	
Badh2-F	ATCCATCTCCGTATCTCT	
Badh2-R	TAGGGTGGTGACTACC	
Hyg-F	GCTGTTATGCGGCCATTGTC	Carrier identification
Hyg-R	GACGTCTGTCGAGAAGTTTC	
Cas9-F	ACCAGACACGAGACGACTAA	
Cas9-R	ATCGGTGCGGGCCTCTTC	
Ubq-q-F	GCTCCGTGGCGGTATCAT	qRT-PCR
Ubq-q-R	CGGCAGTTGACAGCCCTAG	
Hd3a-q-F	GCTCACTATCATCATCCAGCATG	
Hd3a-q-R	CCTTGCTCAGCTATTTAATTGCATAA	
Hd2-q-F	GAACCTATGGCAGCATGTGT	
Hd2-q-R	CGTCATTGCTGCCATTGTT	
Ghd7-q-F	GCTTGAACCCAAACACGG	
Ghd7-q-R	CTCATCTCGGCATAGGCTT	
DTH8-q-F	CAGGAGTGCGTGTCGGAGTT	
DTH8-q-R	GGTCGTCGCCGTTGATGGT	
Pi21-q-F	GGCAAGATCATCAAGGAGATCC	
Pi21-q-R	CTTGGGCTTCTCGCAGTGA	

Table S2. Detailed mutation information of the three selected homozygotes lines.

Line No.	Genotype	Sequence	Mutation Type
**JH-C15**	*Hd2*-JH212	GCTCATCACAACCAAACCGC CGG	**+1 bp**
	Allele1	GCTCATCACAACCAAACTCGC CGG	
	DTH8-JH212	CATCAGCTTCGTTACAGGCG AGG	**-4bp**
	Allele1	CATCAGCTTCGT----GGCG AGG	
	Pi21-JH212	TGCCAAGATCAGGAAGGTCC TGG	**+1bp**
	Allele3	TGCCAAGATCAGGAAGGGTCC TGG	
**JH-C18**	*Hd2*-JH212	GCTCATCACAACCAAACCGC CGG	**-46bp**
	Allele6	GCTCA--------------------------------------AC	
	Ghd7-JH212	CCG GCTGCTGGTCCCATCGACAT	**+1bp**
	Allele1	CCG GCTTGCTGGTCCCATCGACAT	
	DTH8-JH212	CATCAGCTTCGTTACAGGCG AGG	**-1bp**
	Allele1	CATCAGCTTCGTTACA-GCG AGG	
	Pi21-JH212	TGCCAAGATCAGGAAGGTCC TGG	**+1bp**
	Allele1	TGCCAAGATCAGGAAGGTCTC TGG	
**JH-C31**	*Hd2*-JH212	GCTCATCACAACCAAACCGC CGG	**-5bp**
	Allele5	GCTCATCACAAC-----CCGC CGG	
	Pi21-JH212	TGCCAAGATCAGGAAGGTCC TGG	**-1bp**
	Allele4	TGCGATGCCAAGATCAGGAA-TCC TGG	

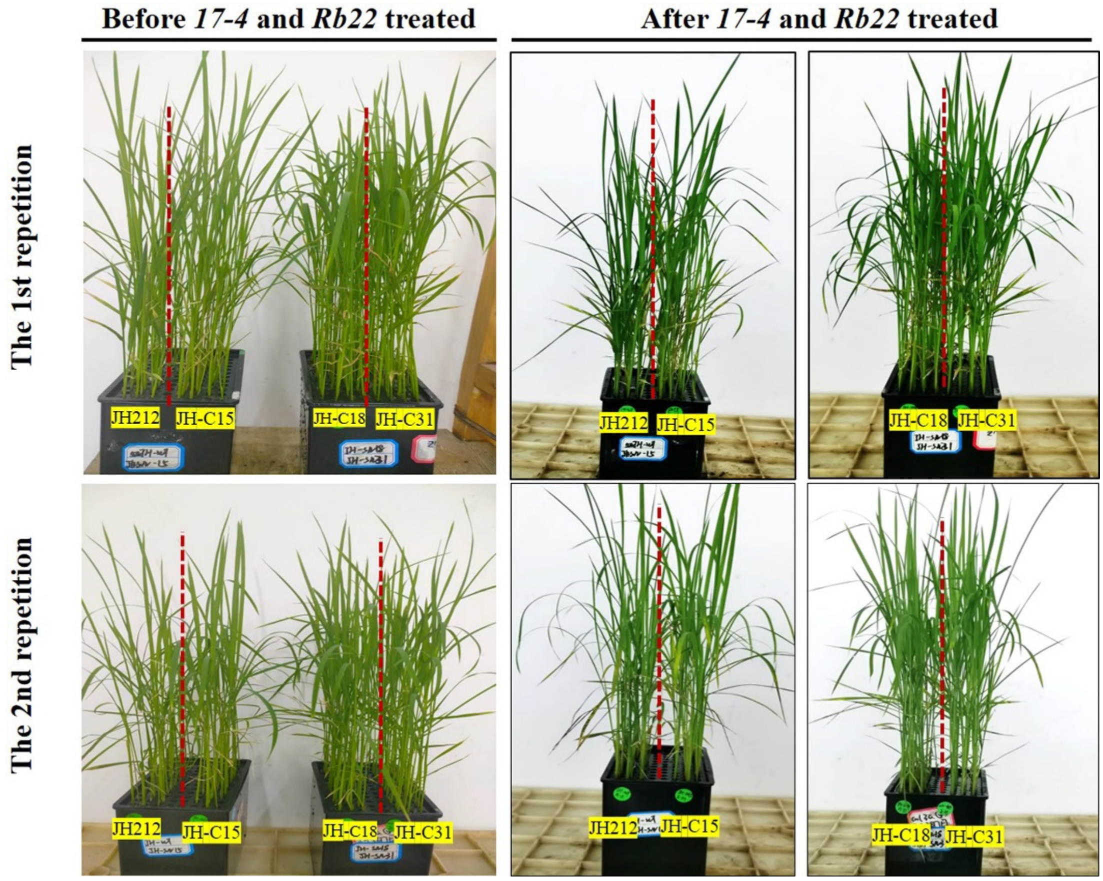

Figure S1. Phenotypic analysis of the T_2 mutant lines and JH212 before and after inoculation with rice blast fungus *17-4* and *Rb22*
